# Supplementary figures and images for: Ammonium hydroxide treatment of Aβ produces an aggregate free solution suitable for biophysical and cell culture characterization
Source: PeerJ. 2013 May 7;1:e73. doi: 10.7717/peerj.73 (PMC3646356; doi:10.7717/peerj.73)

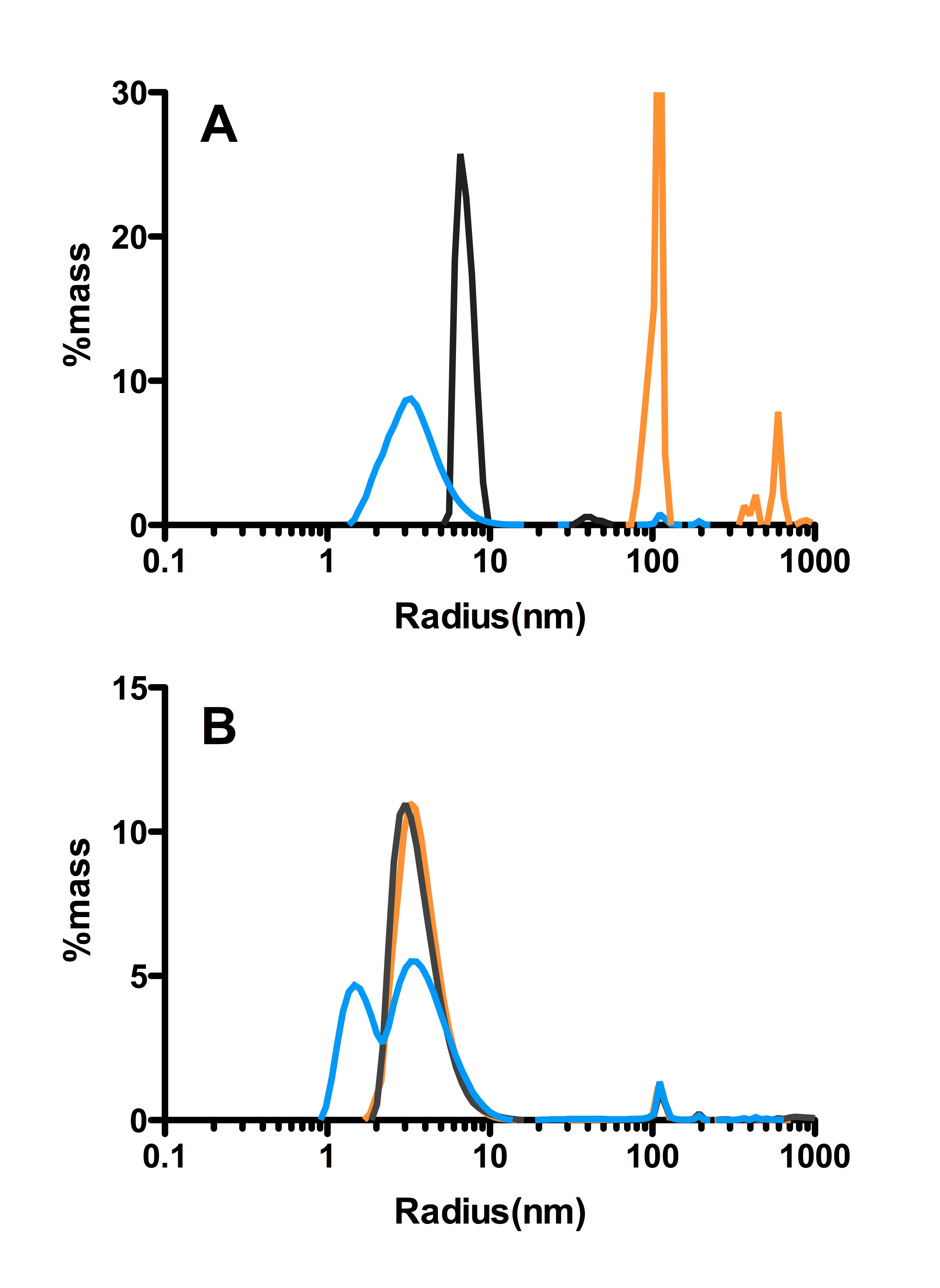

Supplement: Figure S1 — Plotted are the two extremes of the data (smallest radii observed blue, largest distribution, orange) and the most commonly observed distribution for both HFIP (A) and NH4OH (B) treated Aβ (black). These datasets indicate that the HFIP is consistently distributed over a larger range of radii then the NH4OH treated peptide. [file peerj-01-73-s001.jpg]
